# Brain endothelial cells exposure to malaria parasites links type I interferon signalling to antigen presentation, immunoproteasome activation, endothelium disruption, and cellular metabolism

**DOI:** 10.3389/fimmu.2023.1149107

**Published:** 2023-03-13

**Authors:** Abdul Muktadir Shafi, Ákos Végvári, Shanshan Wu Howland, Roman A. Zubarev, Laurent Rénia, Carlos Penha-Gonçalves

**Affiliations:** ^1^ Disease Genetics, Instituto Gulbenkian de Ciência, Oeiras, Portugal; ^2^ Proteomics Biomedicum, Division of Physiological Chemistry I, Department of Medical Biochemistry and Biophysics, Karolinska Institutet, Stockholm, Sweden; ^3^ Division of Physiological Chemistry I, Department of Medical Biochemistry and Biophysics, Karolinska Institutet, Stockholm, Sweden; ^4^ Singapore Immunology Network, Agency for Science, Technology, and Research (A*STAR), Singapore, Singapore; ^5^ A*STAR Infectious Diseases Labs, Agency for Science, Technology, and Research (A*STAR), Singapore, Singapore; ^6^ Lee Kong Chian School of Medicine, Nanyang Technological University, Singapore, Singapore

**Keywords:** cerebral malaria (CM), type - 1 interferons, antigen presentation, endothelial cells, glucose metabolic alterations, proteome, metabolome, malaria

## Abstract

**Introduction:**

Cerebral malaria (CM) lethality is attributable to induction of brain edema induction but the cellular mechanisms involving brain microvascular endothelium in CM pathogenesis are unexplored.

**Results:**

Activation of the STING-INFb-CXCL10 axis in brain endothelial cells (BECs) is a prominent component of the innate immune response in CM development in mouse models. Using a T cell-reporter system, we show that Type 1 IFN signaling in BECs exposed to *Plasmodium berghei*-infected erythrocytes (*PbA*-IE), functionally enhances MHC Class-I antigen presentation through gamma-interferon independent immunoproteasome activation and impacted the proteome functionally related to vesicle trafficking, protein processing/folding and antigen presentation. *In vitro* assays showed that Type 1 IFN signaling and immunoproteasome activation are also involved in the dysfunction of the endothelial barrier through disturbing gene expression in the Wnt/*ß*-catenin signaling pathway. We demonstrate that IE exposure induces a substantial increase in BECs glucose uptake while glycolysis blockade abrogates INFb secretion impairing immunoproteasome activation, antigen presentation and Wnt/*ß*-catenin signaling.

**Discussion:**

Metabolome analysis show that energy demand and production are markedly increased in BECs exposed to IE as revealed by enriched content in glucose and amino acid catabolites. In accordance, glycolysis blockade *in vivo* delayed the clinical onset of CM in mice. Together the results show that increase in glucose uptake upon IE exposure licenses Type 1 IFN signaling and subsequent immunoproteasome activation contributing to enhanced antigen presentation and impairment of endothelial barrier function. This work raises the hypothesis that Type 1 IFN signaling-immunoproteasome induction in BECs contributes to CM pathology and fatality (1) by increasing antigen presentation to cytotoxic CD8+ T cells and (2) by promoting endothelial barrier dysfunction, that likely favor brain vasogenic edema.

## Introduction

Despite the deployment and rollout of state-of-the-art anti-malarial treatments, the malaria death toll reached 627000 cases in 2020 (1). Malaria lethality is largely due to infection by *Plasmodium falciparum* in children and in pregnant women (1). Cerebral malaria (CM) a deadly neurological syndrome affecting mainly children is thought to result from the accumulation of *Plasmodium falciparum-*infected erythrocytes (IE) in the brain vasculature. CM mortality ranges from 15%-20% when effective anti-malarial treatment is available and reached nearly 100% when patients are left untreated (2). Interactions of IE with the brain endothelial cells trigger brain inflammatory responses and disruption of the blood-brain-barrier (BBB) leading to brain swelling, a hallmark of CM pathology (3). However, the molecular mechanisms operating in brain endothelial cells to elicit brain immunopathology and BBB dysfunction are still unresolved.

Studies in *Plasmodium berghei* ANKA (*PbA*) mouse model of experimental CM (ECM) have identified a role for cytotoxic functions in CM-associated immunopathology. CD8 + T cell depletion and perforin or granzyme B genetic ablation have shown that CD8^+^ T cells are critical in ECM pathogenesis. In congruence with pathology observations in the ECM model, CD3^+^ CD8^+^ T cells accumulate in the luminal and perivascular spaces of brain blood vessels in fatal pediatric cerebral malaria cases from Malawi (4, 5). It was recently reported that antigen presentation by class I molecules on brain endothelium dynamically regulates T-cell-mediated neuropathology in ECM (6). Cytotoxic granzyme B, released by activated CD8^+^ T cells when engaging the cognate antigen-MHC class I complex, was detected inside and outside of CD8^+^ T cells lying on brain endothelial cells of infected mice (7).

Innate immune responses involving induction of Type 1 IFN have been identified in multiple stages of malaria infection and are determinants of clinical disease trajectories (8). In particular, Type 1 IFN receptor (IFNAR1) genetic variants were associated with CM in children (9–11), and Type 1 interferon receptor (IFNAR1) signaling was shown to enable effector functions of activated CD8^+^ T cells during ECM development (10). We have previously shown that in the brain parenchyma of *PbA* infected mice, endothelial cells are the main producers of IFNβ, a response determining the recruitment of leucocytes to the brain and ECM immunopathogenic mechanisms (12, 13). However, it remains unexplored whether Type 1 interferon signalling in brain endothelial cells affects endothelial-CD8+T cell interactions or directly impacts BBB permeability.

The involvement of brain endothelial cells in effector phases of CM development is underlined by their ability to acquire and cross-present malaria parasite antigens both in human CM and ECM (4, 14). During *Pb*A infection, epitope-specific CD8+ T cells are induced in the spleen and migrate to the brain before the onset of neurological symptomatology (15). Such epitope-specific CD8+ T cells are elicited by different murine malaria parasite strains but only the immune response triggered by *Pb*A causes neuropathology (15). Notably, *Pb*A infection causes brain microvessels to cross-present parasite antigens, while non-ECM-causing parasites do not (15). The precise mechanisms that are triggered in endothelial cells by exposure to *Pb*A-IE that enable cross-presentation of *Pb*A antigens are unexplored.

In the fatal cases of human CM, brain pathology examination shows either vasogenic edema or cytotoxic edema (16). The tightness of BBB results from intercellular adherence provided by specialized tight junctions and by adherence junctions in the endothelia, which are in part regulated by *ß*-catenin-mediated classical Wnt signaling (17). Alteration of the Wnt signaling leads to the downregulation of the tight junction proteins and increased BBB permeability (17–19). It has been described that tight junction proteins and nuclear *ß*-catenin are decreased in hemorrhagic stroke-mediated and glioblastoma-mediated BBB disruption (17–20). However, it remains unclear whether IE-endothelial cell interactions may target Wnt signaling and promote BBB dysfunction during ECM development. Considering the role of Wnt signaling in the vascular (stroke) and malignancy-medicated BBB disruption, it is likely that Wnt signaling plays similar roles in BBB disruption in the case of ECM and human CM.

For several decades the metabolic relationships between parasites and infected erythrocytes were the focus of intense research leading to a better understanding of parasite biology, identification of anti-malarial drugs, and drug resistance mechanisms (21). However, it was recently proposed that the host’s systemic and cellular metabolism play a role in the response to infection (22, 23). In *Plasmodium chabaudi* AJ murine non-cerebral malaria model, perturbation of glycolysis using 2-deoxy-D-glucose (2-DG) reduced mice survival while administration of glucose showed protective effects (24). On the other hand, using *Pb*A infection, Wang et al. (2018) showed that glycolysis modulation by competitive glucose analog 2DG protects mice from cerebral malaria. Nevertheless, the role of endothelial cell metabolism in cerebral malaria has not been addressed. Endothelial cells are highly glycolytic and glucose is a major contributor to cell energy demand (18). In general, increased glycolysis fluxes have been associated with inflammation while shuttling of glucose towards the OXPHOS and the pentose phosphate pathway dampens inflammation (25).

Here, we investigated the downstream effects of Type 1 interferon signaling in brain endothelial cells (BECs) exposed to *Plasmodium berghei* ANKA infected erythrocytes (*Pb*A-IE) and their interplay with cellular metabolism. We found that the interaction of *Pb*A-IE with BECs elicits a Type 1 interferon response determining enhanced antigen processing, immunoproteasome activation, impaired Wnt*/ß*-catenin pathway signaling, and endothelial barrier disruption. This intrinsic BECs response is licensed by increased glucose consumption which impacts cellular energy metabolism pathways and is implicated in the initiation of ECM pathogenesis.

## Materials and methods

### Animals

All animal procedures were conducted according to national (Portaria 1005/92) and European regulations (European Directive 86/609/CEE) and were approved by the Instituto Gulbenkian de Ciência Ethics Committee (Project number A05.2020) and the Direcção-Geral de Alimentação e Veterinária, the national authority for animal welfare. Mice were housed and bred in the facilities of Instituto Gulbenkian de Ciência. C57BL/6 mice (wild-type) and *Ifnar1^-/-^* (IFNAR KO) mouse strains were obtained from the Instituto Gulbenkian de Ciência facilities. *Ifnβ1* ^−/−^ (IFNβ KO) mouse strain was obtained from the TWINCORE, Centre for Experimental and Clinical Infection Research, Hanover, Germany.

### Murine cerebral malaria model

Wild-type or KO mice were infected with 10^6^ *Pb*A-GFP (intraperitoneal route) obtained from blood-frozen stocks of infected C57BL/6 mice. Parasitemia in infected mice was determined by flow cytometry analysis as GFP+ erythrocytes (LSRFortessa™ X-20 cell analyser, BD Biosciences, and FACSDiVa software version).

Cerebral Malaria progression was evaluated with a 10-point clinical scoring method covering behavioral, visual, gait, and motor functions. Animals were scored on five attributes, observational and task-based, interactions/reflex, cage grasp, visual placing, gait/posture/appearance, and capacity to hold their body weight on a baton. Every attribute was scored from 0 to 2, where 0 represents normal and 2 represents strong impairment (26).

### Brain endothelial cell cultures

Mouse brains were harvested in an ice-cold HBSS medium (Biowest). Brains were minced using a scalpel blade and homogenized by passing through a 23G needle. Homogenates were mixed with an equal volume of 30% dextran (Sigma) and centrifuged at 10000g for 15 minutes. This led to the isolation of brain microvessel pellets, which were suspended in DMEM and passed through a 40u strainer. Brain micro-vessels were retrieved by backflushing with 5 ml of MD131 and were incubated for 2 h at 36.5 °C in an orbital shaker with a digestion cocktail containing 1mg/ml of collagenase type IV (Millipore), 10 μg/ml DNAse (Roche) and 2% FBS. Digested brain microvessels were suspended and cultured in 6, 24 and 48 well-plates in EGM™-2 Endothelial Cell Growth Medium-2 BulletKit™ (LONZA) supplemented with GlutaMax (ThermoFisher) and 10% of FBS, and containing 4 μg/ml puromycin. After 4 days, puromycin containing medium were changed and every 4 days cells were fed EGM™-2 Endothelial Cell Growth Medium-2 BulletKit™ (LONZA) supplemented with GlutaMax (ThermoFisher) and 10% of FBS. When cultures reached 50-80% confluence (~14 days, 95% CD31+ by flow cytometry analysis), the cells were used for experiments.

### Preparation of synchronized infected erythrocytes (*Pb*A-IE)

Blood was collected by cardiac puncture with a heparinized needle from *Pb*A infected mice when parasitemia reached 10% and harvested in 5 ml of RPMI1640 culture medium. Blood was centrifuged at 450g for 8 min and the supernatant was discarded. Erythrocytes were resuspended in RPMI1640 containing 20% FBS in a 250 ml cell culture flask and flushed with a gas-mixture of 5% CO_2_, 5% O_2_ and 90% N_2_. The sealed flasks were incubated in an orbital shaker at 3forring 16 h to synchronize infected erythrocytes in the schizont stage (*Pb*A-IE).

### Cross presentation assays

*In vitro* cross-presentation assays were performed following a previously described protocol (27) briefly described below., Confluent BEC cultures were stimulated with IFNγ (10 ηg/ml), unless stated otherwise. Twenty-four hours after stimulation, cells were stimulated with 2-3 x 10^6^ *Pb*A-IE. After another 24 hours, the medium was withdrawn and the BEC culture was washed with RPMI complete medium and incubated with 6 x 10^4^ reporter cells carrying a TCR transgene that specifically recognizes the PbA peptide (SQLLNAKYL). After overnight incubation, reporter cells were resuspended and added to 96 well filter plate and stained with X-gal. For specific experiments, the medium was supplemented with immunoproteasome inhibitor ONX-0914 (300 nM) or with the glucose analog, 2-DG (10 mM), added 24 hours before cell harvesting.

For *ex-vivo* cross-presentation, brain micro-vessels were isolated from infected and uninfected mice at 6 days after infection (when the wild-type mice show signs of ECM). After isolation, the micro-vessels were passed through a 40 µm strainer and backflushed with 2 ml PBS before incubation with the digestion cocktail (1mg/ml of collagenase type IV (Millipore), 10 μg/ml DNAse (Roche) and 2% FBS) for 1.5 hours. After digestion, microvessels from each brain were distributed per five 96 well filter plates and 3x10^4^ reporter cells were added to each well. 16-18 hours after co-incubation, reporter cells were stained for X-gal.

After staining, the images were captured using a customized stereoscope, assembled with a TCZRS series, Bi-telecentric zoom lenses with motorized control (https://www.opto-e.com/products/8x-bi-telecentric-zoom-lenses-with-motorized-control#Matrix), and RT-mvBF3-2051a, USB3 Vision camera with Sony Pregius CMOS sensor IMX264. Images were captured at 1x magnification with circular illumination. For *ex-vivo* assays, images were processed in ImageJ, and positive events were counted using the find maxima routine. For *in vitro* image processing, event counts were obtained by using an image analysis dedicated script that was implemented to selectively exclude signals from schizonts remnants (red) and only include reporter cells signals (blue).

### Gene expression

BECs were recovered from culture wells. For specific experiments, the medium was supplemented with immunoproteasome inhibitor ONX-0914 (300 nM) or with the glucose analog, 2-Deoxy-d-glucose (2-DG) (10 mM), added 24 h before cell recovery.

cDNA was prepared from each culture well, using a Cells-to-CT kit (Thermo Fisher Scientific). qRT-PCR reactions using standard protocols were performed in ABI QuantStudio-LDA with the following Taqman assays: Mm00440207_m1(Psmb8), Mm00479004_m1 (Psmb9), Mm00479052_g1 (Psmb10), Mm01257559_m1 (Apcdd1), Mm00443610_m1 (Axin2), Mm00727012_s1 (Cld5), Mm00438656_m1 (Edn1), Mm00471902_m1 (Nkd1) and Mm00607939_s1(ACTB), as an internal control. Relative gene expression was quantified using the 2^ΔΔCT^ method.

### Sample preparation for proteomics

Brain endothelial cell cultures from five mouse brains of IFNAR1 KO and wild-type mice were prepared in six-well plates and exposed to 5 x10^6^ *Pb*A-IE/well, in the absence of IFN-γ supplementation. Twenty-four hours after stimulation, un-phagocytosed schizonts were washed out and BMECs were lysed using RIPA lysis buffer followed by precipitation with acetone. For precipitation, 4 x volume chilled acetone was added to the lysate and incubated at 20° overnight. After that, the sample was centrifuged at 15000g for 15 min and protein pellets were isolated and sent for mass spectrometric analysis. Cell pellets were solubilized with 10 µL of 8M urea and 10 µL of 1% Protease MAX (Promega) in 100 mM Tris-HCl, pH 8.5 following sonication in a water bath for 5 min. Following 79 µL of 50 mM Tris-HCl was added together with 1 µL of 100x protease inhibitor cocktail (Roche) before the probe sonicated with VibraCell probe (Sonics & Materials, Inc.) for 40 s, with pulse 2/2, at 20% amplitude. Protein concentration was measured by BCA assay (Thermo Fisher Scientific).

Sample processing and mass spectrometric analysis was performed at the Proteomics Biomedicum (Karolinska Institutet, Stockholm). Briefly, an aliquot of 15 µg sample was transferred to a new tube and equalized with 50 mM Tris-HCl to a total volume of 90 µL. Proteins were reduced with the addition of 3 µL of 250 mM dithiothreitol (Sigma) at 37°C for 45 min and alkylated with 4.5 µL of 500 mM chloroacetamide for 30 min at room temperature in the dark. Sequencing grade trypsin (Promega) was added in an enzyme-to-protein ratio of 1:50 (1.5 µL of 0.2 µg/µL) and digestion was carried out overnight at 37°C. The digestion was stopped with 5 µL cc. formic acid (FA), incubating the solutions at RT for 5 min.

The sample was cleaned on a C18 Hypersep plate with 40 µL bed volume (Thermo Fisher Scientific), and dried using a vacuum concentrator (Eppendorf). Biological samples were labeled with TMT-6plex reagents in random order adding 120 µg TMT-reagent in 30 µL dry acetonitrile (ACN) to each digested sample resolubilized in 70 µL of 50 mM triethylammonium bicarbonate (TEAB) and incubating at room temperature (RT) for 2 h. The labeling reaction was stopped by adding 11 µL of 5% hydroxylamine and incubating at RT for 15 min before combining them in one vial.

### Liquid chromatography-tandem mass spectrometry proteome data acquisition

Peptides were reconstituted in solvent A (2% ACN/0.1% FA) and approximately, two µg samples (2/12 µL) were injected on a 50 cm long EASY-Spray C18 column (Thermo Fisher Scientific) connected to an Ultimate 3000 nanoUPLC system (Thermo Fisher Scientific) using a 90 min long gradient: 4-26% of solvent B (98% ACN/0.1% FA) in 90 min, 26-95% in 5 min, and 95% of solvent B for 5 min at a flow rate of 300 nL/min. Mass spectra were acquired on a Q Exactive HF hybrid quadrupole orbitrap mass spectrometer (Thermo Fisher Scientific) ranging from *m/z* 375 to 1700 at a resolution of R=120,000 (at *m/z* 200) targeting 5x10^6^ ions for a maximum injection time of 80 ms, followed by data-dependent higher-energy collisional dissociation (HCD) fragmentations of precursor ions with a charge state 2+ to 8+, using 45 s dynamic exclusion. The tandem mass spectra of the top 18 precursor ions were acquired with a resolution of R=60,000, targeting 2x10^5^ ions for a maximum injection time of 54 ms, setting quadrupole isolation width to 1.4 Th and normalized collision energy to 33%.

### Proteome data analysis

Acquired raw data files were analyzed using Proteome Discoverer v2.5 (Thermo Fisher Scientific) with Mascot Server v2.5.1 (Matrix Science Ltd., UK) search engine against mouse protein database (SwissProt). A maximum of two missed cleavage sites were allowed for full tryptic digestion, while setting the precursor and the fragment ion mass tolerance to 10 ppm and 0.02 Da, respectively. Carbamidomethylation of cysteine was specified as a fixed modification, while TMT6plex on lysine and N-termini, oxidation on methionine as well as deamidation of asparagine and glutamine were set as dynamic modifications. Initial search results were filtered with 5% FDR using the Percolator node in Proteome Discoverer. Quantification was based on the TMT-reporter ion intensities. Proteome discrimination of three samples per genotype (IFNAR1 KO or wild-type) was obtained using all proteome features with high-dimensional analysis methods; principal component analysis (PCA) and orthogonal partial least squares discriminant analysis (OPLS-DA). Volcano plots were used to identify proteome features differentially represented in IFNAR1 KO BEC samples.

### Multiple pathway targeted analysis

Cell pellets were pre-extracted and homogenized by the addition of 1000 µL of MeOH: H2O (4:1), in the Cryolys Precellys 24 sample Homogenizer (2 x 20 seconds at 10000 rpm, Bertin Technologies, Rockville, MD, US) with ceramic beads. The bead beater was air-cooled down at a flow rate of 110 L/min at 6 bar. Homogenized extracts were centrifuged for 15 minutes at 4000 g at 4°C (Hermle, Gosheim, Germany). The resulting supernatant was collected and evaporated to dryness in a vacuum concentrator (LabConco, Missouri, US). Dried sample extracts were resuspended in MeOH: H2O (4:1, v/v) according to the total protein content.

Media samples (25 µL) were extracted with 100 µL of MeOH. Homogenized extracts were centrifuged for 15 minutes at 4000 g at 4°C (Hermle, Gosheim, Germany). The resulting supernatant was collected and injected into the LC-MS system. BCA Protein Assay Kit (Thermo Scientific, Massachusetts, US) was used to measure (A562nm) total protein concentration (Hidex, Turku, Finland).

Extracted samples were analyzed by Hydrophilic Interaction Liquid Chromatography coupled to tandem mass spectrometry (HILIC - MS/MS)1,2 in both positive and negative ionization modes using a 6495 triple quadrupole system (QqQ) interfaced with 1290 UHPLC system (Agilent Technologies). Pooled QC samples (representative of the entire sample set) were analyzed periodically (every 4 samples) throughout the overall analytical run in order to assess the quality of the data, correct the signal intensity drift and remove the peaks with poor reproducibility (CV > 30%) (28–30).

### Flow cytometry and IFN-beta measurements

After the confluence of the BECs, cells were detached using TrypLE™ Express Enzyme (1X), no phenol red, and stained with anti-CD31 (APC, Anti-Mouse) (BD Pharmingen™) MHCI (A488, Anti-Mouse) and propidium iodide as per the standard flow cytometry protocol and analyzed thereafter (LSRFortessa™ X-20 cell analyzer, BD Biosciences, and FACSDiVa software version). IFN-Beta was measured in the supernatant of BECs cultures using high sensitivity VeriKine-HS Mouse Interferon-Beta ELISA Kit (cat. nr.: 42410) from PBL Assay Science. Procedures followed the manufacture protocol for tissue culture medium using 50 uL of supernatant per sample.

### Measurements of transendothelial electrical resistance TEER

BECs were grown to confluence in VWR Tissue Culture Plate Inserts 24 well PET membrane, 8µm. Trans-endothelial electric resistance (TEER) was recorded using Millicell ERS-2 Voltohmmeter. The electric resistance plateau corresponding to the establishment of an endothelial is reached on the range 200-300 Ohm/cm2. A detailed description of this method has been published elsewhere (31). Once a stable measurement of resistance was achieved, BECs in the inserts were exposed to 1.5 x 10^6^ freshly prepared *Pb*A-IE or left unexposed and treated or not with GSK 3 inhibitor CHIR-99021(1 µM) for 24 h. Electric resistance was monitored in each plate well at selected time points and expressed as percent of the respective plateau resistance measurement.

### Glycolysis inhibition and glucose measurements

For *in vivo* glycolysis inhibition experiments mice were injected intra-peritoneally with 2-DG (800 mg/kg) at day 4 four days after infection with *Pb*A-IE. The timing of injection was chosen according to evidence that IFNb is expressed in the brain by day 4 after infection (13).

For measurement of glucose uptake, BECs were cultured in 48 well culture plates and exposed to 1.5 to 3 x 10^6^ schizonts for 24 h in presence of absence of 2-DG (10 mM). The supernatant was collected at specific time points during this period and centrifuged at 10000g for 2 min. Glucose was measured by @Accucheck glucose measurement kit. To ascertain whether glucose consumption was not attributable to *Pb*A-IE a set of experiments, where after 24-h exposure of the BECs to *Pb*A-IE, the supernatant containing IE was discarded and BECs properly washed to avoid any leftover of the IE. Thereafter fresh medium was dispensed in each well for 24 h and glucose was measured in the collected media (**Figure S3**).

### Statistics

Calculations of ANOVA tests, area under the curve (AUC), and Log-rank (Mantel-Cox) test were performed using Prism 9 for macOS from GraphPad Software, LLC.

## Results

### Type 1 interferon signaling controls endothelial antigen presentation and immunoproteasome induction

**Figure 1:**
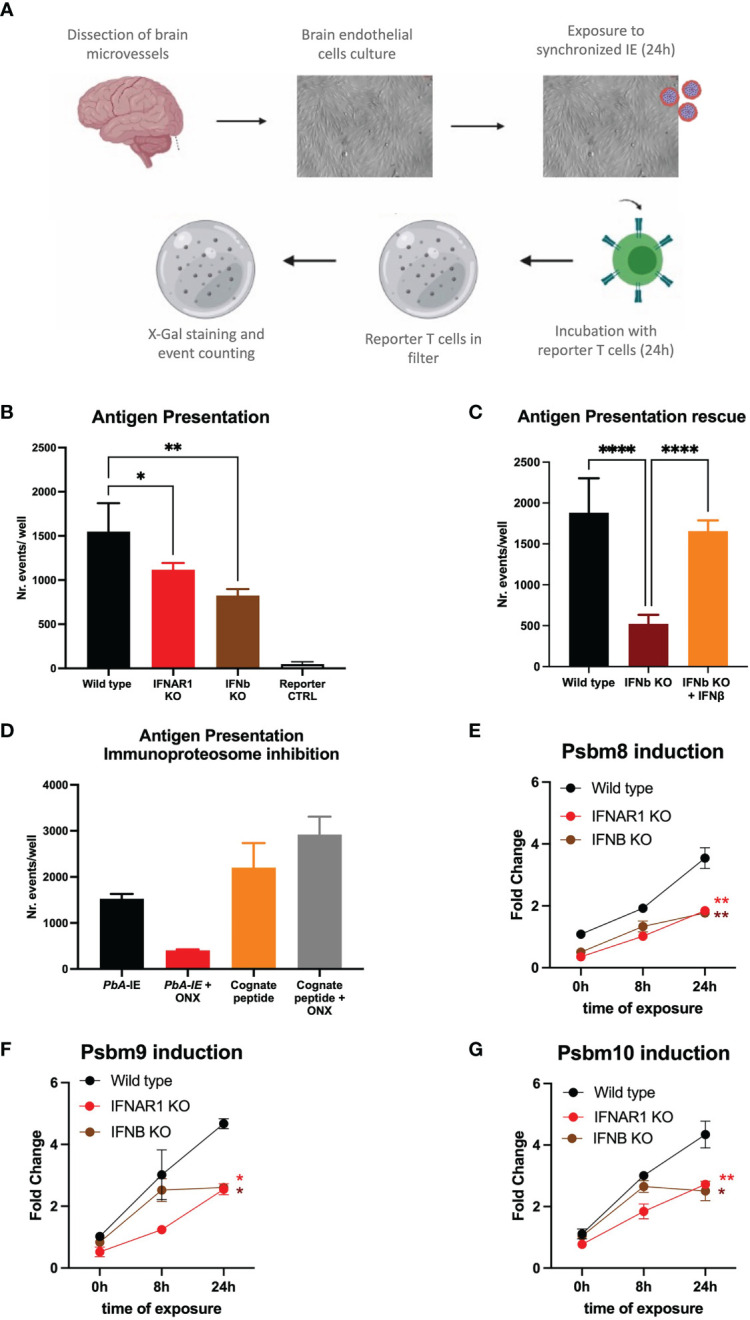
IFNAR1 signaling is a determinant of immunoproteasome-dependent antigen presentation in brain endothelial cells exposed to PbA-IE. **(A)** Diagram of *in vitro* antigen presentation assay depicting primary BEC cultured in presence of interferon-gamma (24h), exposed to synchronized *PbA*-IE (24h) and incubated with *PbA* antigen-specific reporter T-cell line that are subsequently stained to count antigen presentation events. **(B)** Antigen presentation events counts in BEC cultures from wild-type IFNAR1 and IFNB KO mice. **(C)** Results of two independent experiments counting antigen presentation events in BEC cultures from wild-type and IFNB KO mice in the presence or absence of IFNβ supplementation (80-120 µg/ml). **(D)** Results of two independent experiments counting antigen presentation events after exposing wild-type BEC cultures to *PbA*-IE or to reporter cell cognate peptide 10 µM with no supplementation with IFN-γ and in the presence or absence of immunoproteasome inhibitor ONX-0914 (300 nM)(ONX). Kinetics of *Psbm8* **(E)**, *Psbm9* **(F)**, and *Psbm10* **(G)** gene expression quantified by qPCR (fold change represents 2^ΔΔCT^ relative to wild-type at 0h) and showing induction in BECS of wild-type, IFNAR1 KO and IFNB KO mice exposed to *PbA*-IE, in absence of IFN-γ. Statistics: P-value of ANOVA tests of antigen presentation results (black stars) or ANOVA tests of AUC in gene expression induction in IFNAR1 vs wild-type (red stars) and IFNB versus wild-type (brown stars). (*; p<0.05, **; p<0.01, ****; p <0.0001).

Interactions of brain endothelial cells (BECs) with IE are key in CM pathogenesis. To dissect molecular events resulting from these interactions we used an *in vitro* system where endothelial cell cultures derived from mouse brain vessels are exposed to *PbA-infected* erythrocytes (*Pb*A-IE). We have previously shown that this experimental system allows activation of cytoplasmic innate receptors and subsequent induction of Type I IFN responses, suggesting it entails delivery of malaria antigens in BECs cytoplasm (13, 31). Malaria antigen cross-presentation was studied following a described method that uses a reporter T cell line recognizing the SQLLNAKYL *Pb*A peptide (derived from Glideosome-associated protein 50) in the context of mouse MHC-b haplotype (H-2D^b^) (**Figure 1A**) (27). We quantified events of cross-presentation to reporter T cells in primary BEC cultures. We found that BECs from IFNAR1 and IFNb KO mice show reduced efficiency in presenting malaria antigens (**Figure 1B**). Medium supplementation with IFNβ rescued antigen presentation efficiency of IFNB KO BECs (**Figure 1C**), indicating that Type I IFN signaling through IFNAR1 is a determinant of malaria antigen presentation by BECs.

In response to inflammation or infection, proteolytic subunits of constitutive proteasome are replaced by immunoproteasome subunits Psmb8, Psmb9 and Psmb10 that are more efficient in processing endocytosed antigens for subsequent MHC I-restricted cross-presentation (32). We examined *Plasmodium* antigen presentation by BECs under treatment with ONX-0914 (PR-957) (ONX), a selective inhibitor of *Psmb8* (33). We found that incubation with ONX 300 nM did not affect antigen presentation of MHC-I loaded cognate peptide but sharply decreased antigen presentation upon exposure to *PbA*-IE (**Figure 1D**). It is well-known that immunoproteasome subunits synthesis is induced by interferon-gamma (IFN-γ) and we asked whether immunoproteasome induction in BECs was controlled by Type 1 IFN signaling, in the absence of IFN-γ supplementation. We found that gene expression of three immunoproteasome subunits (*Psbm8*, *Psbm9*, and *Psbm10*) is induced in the course of exposure to *Pb*A-IE in wild-type BECs but was significantly lower in IFNAR1 KO and IFNb KO BECs (**Figures 1E, F**). Together these findings unveil that IFNAR1 signaling in BECs exposed to *Pb*A-IE operates the induction of immunoproteasome subunits synthesis that in turn contributes to increased efficiency of intracellular *Plasmodium* antigens cross-presentation.

### Type 1 interferon signaling impacts protein processing and surface MHC class I molecules availability

**Figure 2:**
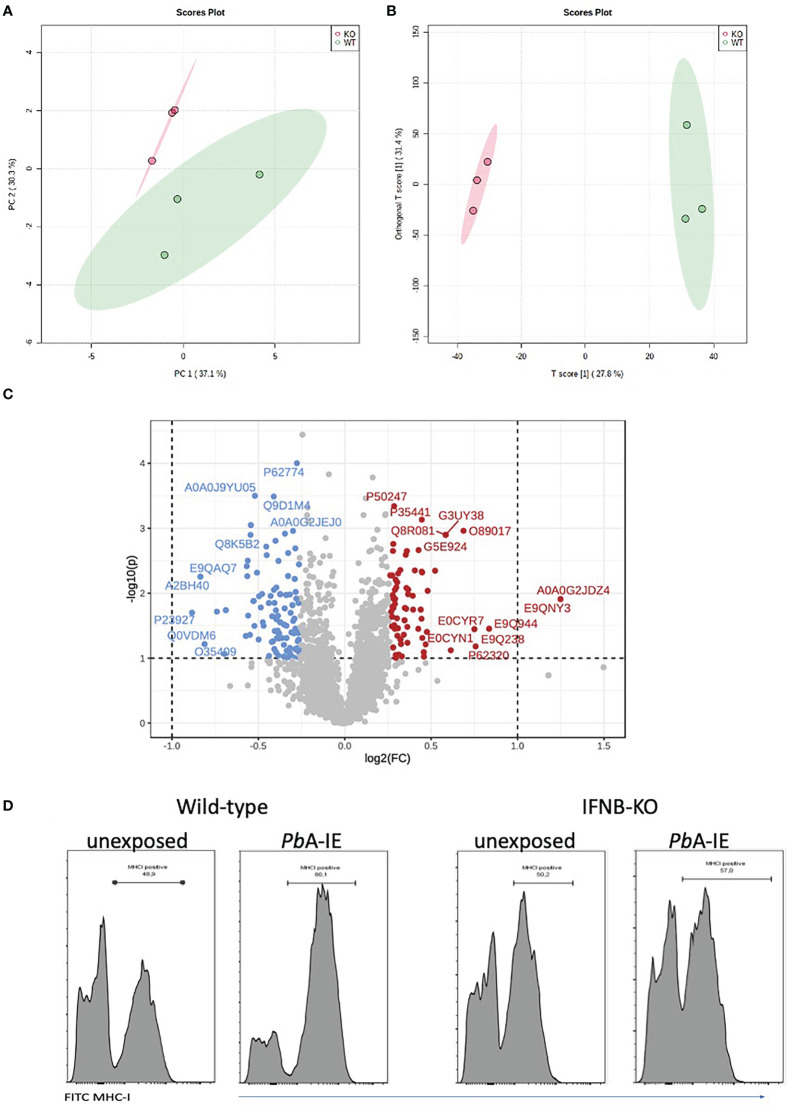
IFNAR1 signaling impact the proteome and MHC class I expression in BECs exposed to *Pb*A-IE. Proteome discrimination analysis of BECs cultures from IFNAR1KO and wild-type mice exposed to *Pb*A-IE. Principal component analysis score plot (PC1versus PC2) **(A)** and orthogonal partial least squares discriminant analysis (OPLS-DA) score plot **(B)** of all detected protein features in BECs samples (wild-type in green, IFNAR1 KO in red); explained variance is shown in brackets in axes titles. **(C)** Volcano plot highlighting differentially expressed protein features and showing fold change in IFNAR KO relative to wild-type (FC) log(2)FC > 1.2 (log2(FC) > 0.18, red; log2(FC) < - 0.18, blue) and t-test significance level, P<0.05. Differentially expressed protein features are identified by the respective SwissProt database reference. Proteome analysis is based on three independent samples per genotype, each sample representing BECs obtained from a pool of five mouse brains. **(D)** Flow cytometry histogram plots of surface staining of MHC class I molecules in BEC of IFNB KO and wild-type mice, cultured in the absence or presence of *Pb*A-IE (24h). Results are representative of 2 independent experiments.

Given that Type 1 IFN signaling was inducing the antigen processing machinery we asked whether it was also affecting the endothelial cell proteome. We performed a comparative proteome analysis in triplicate-independent samples of wild-type and IFNAR1 KO BECs exposed to *Pb*A-IE for 24 h, in the absence of IFN-γ supplementation. Notably, high-dimensional analyses (principal component analysis and orthogonal partial least squares discriminant analysis) of all mouse proteome features identified two clusters clearly distinguished according to the IFNAR1 genotype (**Figures 2A, B**). Further, 207 protein features were identified as differentially expressed in wild-type and IFNAR1 KO BECs (univariate p value<0.05; FC >1.2) (**Figure 2C**). A more stringent selection of differentially expressed protein features (P<0.01) identified 34 proteins that were functionally grouped (**Table S1**). These analyses show that Type 1 IFN signaling significantly impacts endothelial proteome upon exposure to *Pb*A-IE and notably controls the expression of proteins related to antigen presentation, protein processing, and vesicle trafficking. Furthermore, flow cytometry analysis showed that IFNb KO BECs are not able to increase expression of surface expression of MHC class I molecules upon exposure to *Pb*A-IE (**Figure 2D**). These results indicate that autocrine/paracrine Type I IFN secretion and subsequent signaling through IFNAR1 enhances activation of antigen presentation pathway and availability of MHC class I molecules for antigen presentation in BECs exposed to IE. These data uncovered that Type 1 IFN signaling enhances BECs’ efficiency in presenting intracellular parasite antigens to CD8^+^T cells by acting on immunoproteasome activation, protein processing, vesicle trafficking and MHC class I molecules availability.

### IFNAR1 signaling and immunoproteasome contribute to endothelial barrier disruption

**Figure 3:**
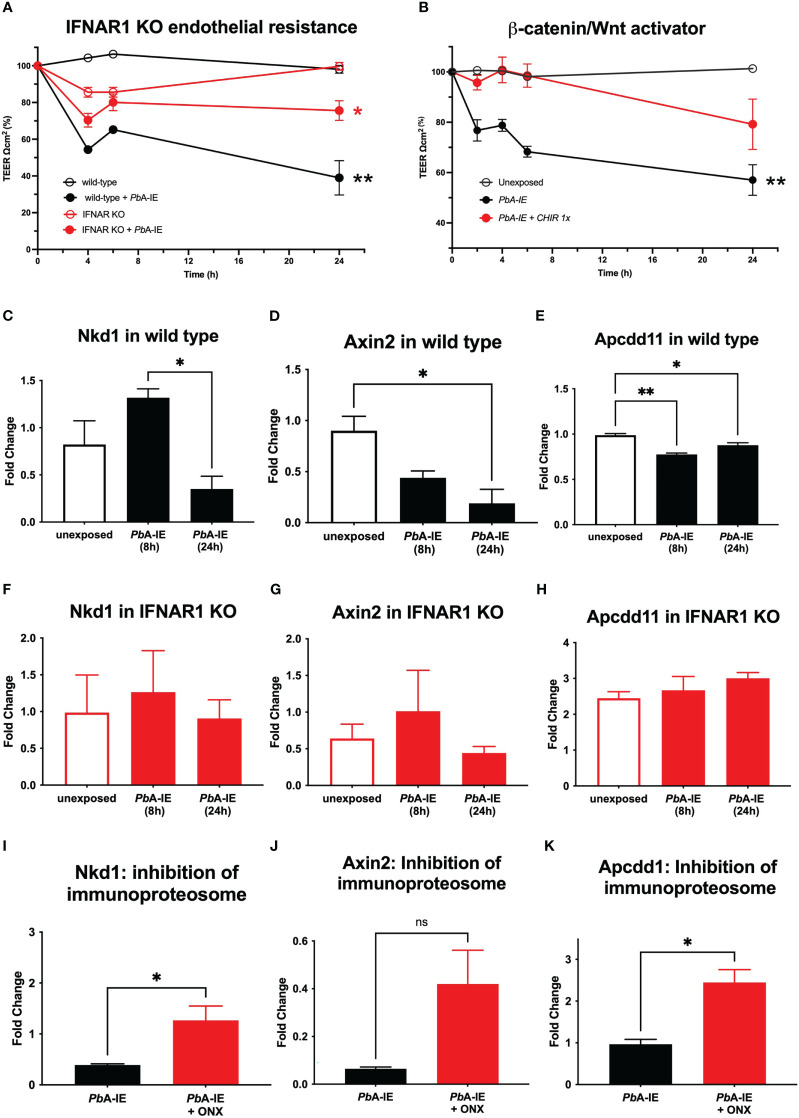
IFNAR signaling a lead to disruption of the brain endothelial barrier through immunoproteosome-dependent Wnt/*ß*-catenin signaling disturbances. Endothelial barrier integrity was monitored by electric resistance measurements of transwell BEC cultures (TEER) at the indicated time points after exposure or not to *Pb*A-IE **(A, B)**. TEER kinetics in wild-type, IFNAR1 KO BECs **(A)**, or in wild-type BECs in the presence or absence of (GSK) 3 inhibitor CHIR-99021(1 µM) **(B)**. Gene expression of *ß*-catenin target genes *Nkdd1, Axin2*, and *Apcdd1* was quantified by qPCR in wild-type **(C–E)** and IFNAR1 KO BECs **(F–H)** 24 h after exposure to *Pb*A-IE in presence or absence of immunoproteasome inhibitor ONX-0914 (ONX) (300 nM) **(I–K)**. Relative quantification of gene expression is represented as fold change (2^ΔΔCT^) using unexposed BECs as controls. Statistics: Significant results of ANOVA tests of AUC TEER in wild-type BECs exposed to PbA-IE versus unexposed (black stars) and in IFNAR1 KO BECs exposed versus unexposed (red stars). Significant results of pairwise comparisons in ANOVA tests in gene expression experiments are shown (*; p<0.05, **; p<0.01).

Type I IFN signaling in CD8+ T cells plays a critical role in cytotoxic functions that lead to BBB disruption during ECM development (10). Exploring another angle, we examined whether Type I IFN signaling in BECs intrinsically contributed to the disruption of the brain endothelial barrier. BECs were cultured in confluence until increasing trans-endothelial electric resistance (TEER) reached a high plateau that corresponds to the establishment of an endothelial barrier and were challenged with *Pb*A-IE (31). In wild-type BECs, TEER falls 60% by 24h after exposure to *Pb*A-IE indicating disruption of the endothelial barrier. In contrast, TEER showed a reduced decline in IFNAR1 KO BECs, indicating that the absence of IFNAR1 signaling offers partial resistance to endothelial barrier disruption upon *Pb*A-IE exposure (**Figure 3A**). Proteome analysis corroborated that IFNAR1 signaling is associated with downregulation of a noticeable number of proteins related to the dynamics of cytoskeleton organization that influence cell shape and motility possibly contributing to endothelial barrier disruption (**Table S1**).

It is known that intracellular Wnt/*ß*-catenin pathway disturbances are associated with BBB dysfunction and are involved in the pathogenesis of several brain diseases (20). We observed that disruption of the endothelial barrier induced by *PbA*-IE was prevented in BECs treated with CHIR-99021 (**Figure 3B**). This treatment effectively activates Wnt signaling by inhibiting glycogen synthase kinase (GSK) 3 which takes part of the *ß*-catenin destruction complex. Further, we found that expression of Wnt/*ß*-catenin signaling pathway genes (*Nkd1*, *Axin2*, and *Apcdd1*) was down-regulated in wild-type BECs upon exposure to *Pb*A-IE suggesting that Wnt/*ß*-catenin signaling was impaired (**Figures 3C–E**). On the other hand, the expression of those target genes was not significantly disturbed in IFNAR1 KO BECs (**Figures 3F–H**). These results suggested that upon *Pb*A-IE challenge IFNAR1 signaling contribute to endothelial barrier dysfunction by impairing Wnt/*ß*-catenin signaling.

Next, we explored the possibility that impaired transcription of Wnt/*ß*-catenin target genes resulted from increased immunoproteasome activation, which is known to promote the destruction of ubiquitinated *ß*-catenin (34). We found that the immunoproteasome inhibitor (ONX) counteracts the downregulation of Wnt/*ß*-catenin signaling pathway genes induced by *Pb*A-IE although this effect did not reach statistical significance in the case of the *Axin2* gene (**Figures 3I–K**). This suggests that immunoproteasome activation disturbs Wnt signaling possibly by acting on ubiquitinated *ß*-catenin. Cohesively, these findings unveil a mechanism of brain endothelium barrier disruption elicited by *PbA*-IE that involves Type 1 interferon-mediated immunoproteasome activity and consequent disturbance of WTN/B-catenin signaling.

Interestingly, gene expression of claudin 5, a tight junction protein that regulates paracellular ionic selectivity, was strongly down-regulated in wild-type BECs exposed to *Pb*A-IE (**Figure S1A**). This effect was not restored in absence of IFNAR1 signaling neither was affected by immunoproteasome inhibition (**Figures S1B, C**). Nevertheless, claudin 5 gene expression in the brains of infected mice was reduced as compared to non-infected animals (**Figure S1D**). On the other hand, proteins that are part of adherens-type cell-cell and cell-matrix junctions (e.g. thrombospondin-1, afadin, plectin, and phosphoglucomutase-like protein 5) are down-regulated by Type 1 IFN signaling in BECs exposed to *Pb*A-IE (**Table S1**). These results indicate that cell-cell junction disturbances evoked by *Pb*A-IE are in part dependent on Type 1 IFN signaling but suggest that additional mechanisms may play a role in endothelial barrier disruption caused by *Pb*A-IE.

### Glucose consumption promotes type 1 interferon and associated responses in BECs exposed to *Pb*A-IE

**Figure 4:**
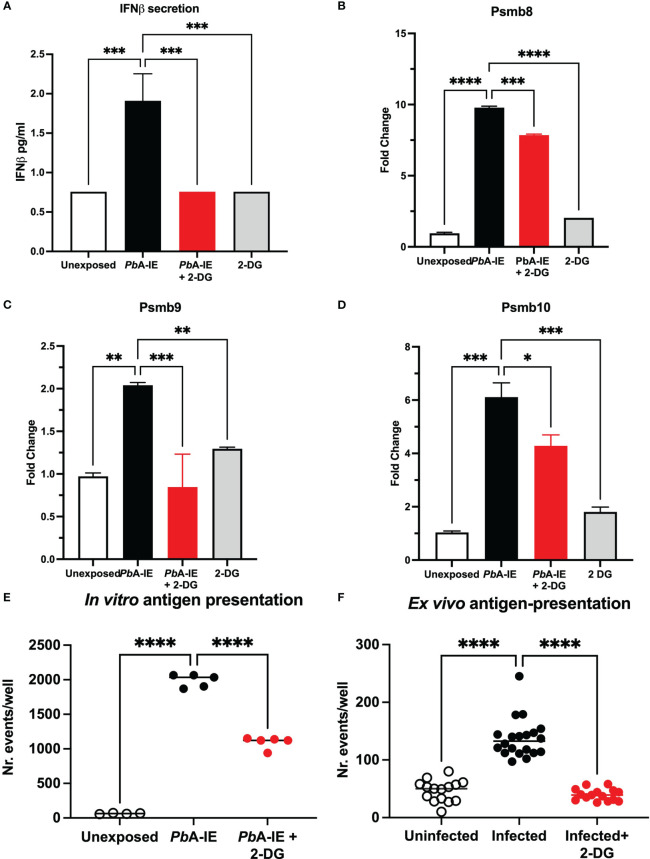
Glucose metabolism inhibition impairs IFN-ß secretion, immunoproteasome induction and antigen presentation in brain endothelial cells upon contact with *Pb*A-IE. Effects of glycolysis inhibition in BEC cultures in presence 2-DG (10 mM) measured 24 h after exposure to *Pb*A-IE. **(A)** Quantification of secreted IFN-ß in culture supernatants of BECs exposed or not to PbA-IE in the presence or absence of 2-DG (lower limit of quantitation 0.94 pg/mL). **(B–D)** Gene expression of immunoproteasome genes *Psmb8*, *Psmb9*, and *Psmb10* in BECs in the indicated conditions. Cumulative results of independent experiments are represented as fold change (2^ΔΔCT^) using unexposed BECs as controls. Antigen presentation assays in BEC cultures unexposed and exposed to *Pb*A-IE *in vitro* **(E)** and in BECs from uninfected and infected mice **(F)**, treated or not with 2-DG. Significant results of pairwise comparisons in ANOVA tests are shown (*; p<0.05, **; p<0.01, ***; p<0.001, ****; p <0.0001).

Inflammatory stimuli have been shown to enhance glycolysis in endothelial cells (25). We investigated whether glycolysis controls Type 1 IFN response in BECs exposed to *Pb*A-IE. To this end, we made use of glucose analog 2-DG, that competitively inhibits the production of glucose-6-phosphate from glucose, thereby blocking glycolysis. Secreted IFN-ß was barely detected in BEC culture supernatants but was increased upon exposure to *Pb*A-IE. We found that adding 2-DG to BEC cultures abrogates IFN-ß secretion (**Figure 4A**) in *Pb*A-IE exposed cells, presumably impairing Type 1 IFN signaling-associated responses. Indeed, we found that induction of immunoproteasome genes *Psmb8*, *Psmb9*, and *Psmb10* was decreased by 2-DG when BECs were exposed to *Pb*A-IE (**Figures 4B–D**). Likewise, the efficiency of antigen presentation *in vitro* was affected when BEC cultures were incubated with 2-DG (**Figure 4E**) which did not affect the ability to efficiently present cognate peptides loaded on surface MHC class I molecules (**Figure S2A**). Notably, we performed *ex vivo* antigen presentation experiments using BECs from *Pb*A-IE infected mice and found that enhancement of antigen presentation by BECs exposed to *in vivo* infection was abrogated in 2-DG treated mice (**Figure 4F**). In addition, we found that down-regulation of *Wnt/ß*-catenin target genes in *Pb*A-IE exposed BECs was reverted at some extent in presence of 2-DG, although we cannot exclude that inhibition of glycolysis by 2-DG exerted a direct effect in inducing expression of these genes (**Figure S2B**). These results indicate that glucose metabolization is needed to license the observed Type 1 IFN signaling downstream effects, namely enhancement of antigen presentation and immunoproteasome activation.

### Metabolism re-programming in BECs exposed to *Pb*A-IE

**Figure 5:**
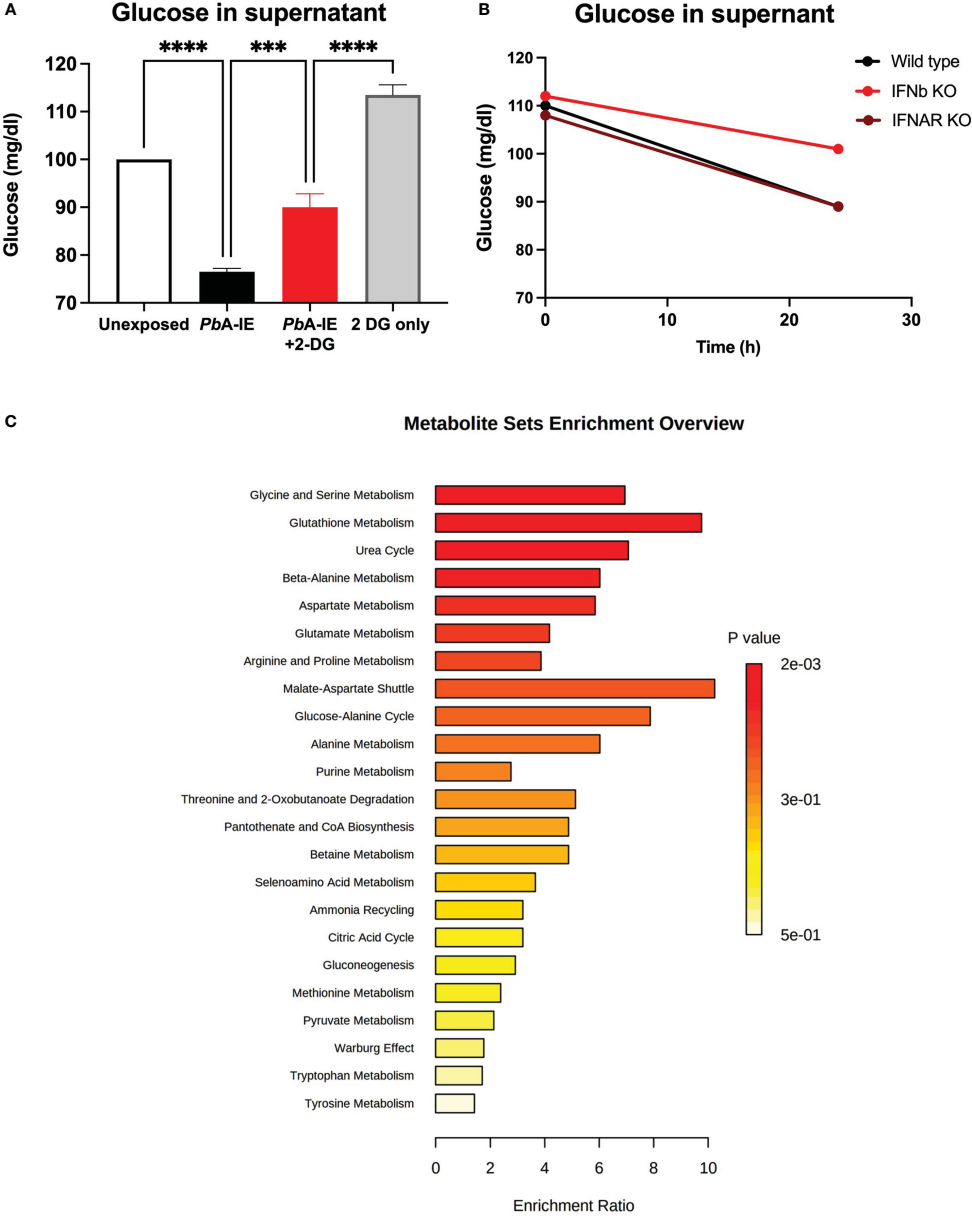
Glucose uptake and metabolism in brain endothelial cells exposed to *Pb*A-IE. Measurement of glucose in supernatants of **(A)** BECs cultures after 24 h of exposure to *Pb*A-IE in the presence or absence of 2-DG (10 mM) and **(B)** in cultures from wild-type, IFNAR1 KO and IFNB KO BECs in presence of 2-DG. **(C)** Multiple pathway targeted analysis showing enrichment ratio (P-value) of metabolic pathways in wild-type BECs exposed to *Pb*A-IE as compared to unexposed BECs (MetaboAnalyst 5.0). Significant results of pairwise comparisons in ANOVA tests are shown (***; p<0.001, ****; p <0.0001).

To ascertain the effects of BEC exposure to *Pb*A-IE on glucose consumption we made use of 2-DG glycolysis inhibition and measured the amount of glucose in the culture supernatant as an indirect readout of glucose uptake by BECs. We found that 24h exposure of BECs to *Pb*A-IE resulted in depletion of glucose amounts in the cell culture medium, an effect significantly inhibited by 2-DG blocking (**Figures 5A** and **S3A**). Type 1 IFN signaling responses did not affect glucose uptake as BECs from IFNAR1 and IFNb KO mice exposed to *Pb*A-IE also showed glucose depletion from the cell culture medium (**Figure 5B**). Nevertheless, proteome analysis in BECs exposed to PbA-IE shown that in the presence of IFNAR1 signaling, enzymes related to ATP metabolism namely, ATP synthase subunit gamma and adenine phosphoribosyltransferase, are up-regulated which critically contributes to AMP synthesis through the adenine salvage pathway (**Table S1**).

These observations led us to perform multiple metabolic pathway targeted analysis comparing BECs exposed or not to *PbA-IE*. We found that exposure to *PbA*-IE affected multiple pathways related to glucose metabolism which correlated with suggested increased energy demand and production (**Figure 5C**) with a significant increase of individual metabolites such as pyruvate, lactate and metabolites from TCA (**Table S2**). We also noted that several acylcarnitines were increased suggesting that the transport of fatty acids into mitochondria for energy production is enhanced following exposure to *PbA-IE*.

In addition, we observed that amino acid metabolism was affected in multiple pathways (**Figure 5C**). Several amino acids (isoleucine, alanine, and aspartate) and intermediate metabolites related to amino acid catabolism (e.g. glutarate and 4-acetamidobutanoate) showed increased amounts in BECs exposed to *PbA-IE*. Unexpectedly, methylated amino acid derivatives such as trimethyllysine, dimethylarginine, and betaine (**Table S2**) were also increased. Together with the observation that adenosylhomocysteinase is reduced in wild-type BECs exposed to *PbA*-IE (**Table S1**), these results suggest that the activated methyl cycle was affected. Altogether these findings provide evidence that exposure to *Pb*A-IE strongly increased glucose uptake in BECs and suggest that cellular downstream effects associated with Type 1 IFN signaling are dependent on increased glucose consumption. Nevertheless, the precise impact of exposure of BECs to *PbA-IE* in specific metabolic pathways requires detailed studies namely on glucose metabolic fluxes.

### Glucose metabolism blockade in infected mice

**Figure 6:**
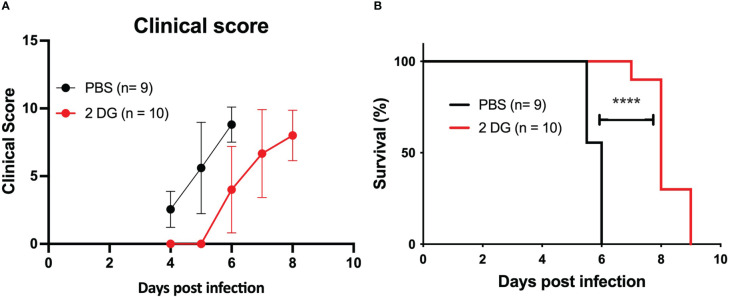
2-DG treatment increases the survival of infected mice. Clinical scoring **(A)** and survival curves **(B)** of wild-type mice infected with *Pb*A-IE and treated (2-DG) or not (PBS) with 2-DG (800 mg/kg) at day 4 post-infection. Log-rank (Mantel-Cox) test **(D)** (****; p <0.0001).

Glucose metabolism mediates disease tolerance and resistance in cerebral malaria (22). We tested whether the development of CM in the *PbA* infection mouse model was affected by the inhibition of glucose uptake. We treated infected mice with one 2-DG injection (800 mg/kg) on day 4 when mouse BECs start expressing IFNβ and clinical symptoms of cerebral malaria are still incipient (13). Daily clinical scoring of cerebral malaria revealed that 2-DG treatment delayed the clinical onset of neurological symptoms (**Figure 6A**) and increased the survival time of infected mice compared to non-treated mice (**Figure 6B**). As observed by others (35), 2-DG treatment immediately increased glucose blood levels indicating that cellular glucose uptake was reduced but it did not reduce the amount of *Pb*A-IE as ascertained by the parasitemia level (**Figure S3B**). This suggests that 2-DG treatment during infection exerted a protective effect on the brain tissue.

In sum, these results indicate that increased glucose uptake and glycolysis elicited by *Pb*A-IE exposure affects Type 1 IFN mediated responses in BECs suggesting that beneficial effects of glucose blockade in the initiation of clinical ECM may be linked to pathogenesis mechanisms operated by Type 1 IFN in BEC endothelium early in infection (13).

## Discussion

This work links Type 1 IFN signaling in BECs exposed to *PbA-*IE to downstream cellular effects that eventually result in BBB damage. We found that Type 1 IFN signalling increased the expression of immunoproteasome components and MHC class I molecules and, in interrelated association enhanced the antigen presentation of malaria parasite antigens to CD8+ T cells. In the course of the disease, this effect likely contributes to the cytotoxic effector functions of activated CD8+ T cells that target and damage the brain microvascular endothelium. In addition, immunoproteasome activation upon exposure to *PbA*-IE, conditioned disturbances of Wnt signaling that in turn contributed to destabilize the endothelial barrier. This likely operates through imposing dysfunction on intercellular tight junctions, the first step towards increased BBB permeability which typically occurs in CM.

Remarkably, we found that increased glucose consumption in *PbA-*IE exposed BECs precedes Type 1 interferon response and its downstream effects. This uncovered that BEC’s innate immunity activation by *PbA-*IE is entangled with cellular metabolic alterations. Accordingly, we found that clinical ECM onset was delayed by blockade of glucose consumption, suggesting that increased glucose consumption is a precipitating factor in ECM pathogenesis that acts by licensing innate immunity activation of brain endothelial cells.

The pathogenic role of endothelium activation in the context of the innate immune response during malaria infection is catching increasing attention (12). A large body of evidence links brain endothelium activation to the enhancement of adhesive properties underlying IE sequestration in the brain. ICAM-1, CD36, and EPCR have been identified as adhesion molecules that act as counterparts of parasite molecules exported to the IE surface (36). The expression of endothelial adhesion molecules is induced by TNF during malaria infection, suggesting that the inflammatory milieu promotes changes in endothelium adhesivity (37, 38). IE sequestration has been considered a key event in CM pathology promoting local vascular inflammation and brain microcirculatory alterations (39). Nevertheless, it is also clear that brain immunopathology events that lead to BBB disruption and brain edema play a determinant role in many CM cases (40). Our results support the notion that BECs activation during malaria infection does not require IE cytoadherence and drives engagement of activated antigen-specific immune cells by presenting parasite antigens leading to brain immunopathology.

Here, we investigated the effects of Type 1 IFN signaling that promote innate immunity functions in BECs and contribute to ECM pathogenesis. Our approach unveiled the activation of cellular immunity mechanisms that promote antigen cross-presentation and are dependent on the autocrine action of IFN-ß. We have previously shown that IFNβ production by brain endothelial cells is a determinant of ECM development (13) and that CD8+ T cells required IFNAR1 expression to exert pathological effects in the brain and provoke ECM (10). On the other hand, it has been demonstrated that BECs are induced to cross-present cytosolic malaria antigens to CD8+ T cells *in vitro* and *ex vivo* (15, 27, 41). Together, those results convey the notion that BEC’s Type 1 IFN response to IE autonomously promotes engagement of cytotoxic CD8+ T cells at two distinct levels namely, cross-presentation of parasite antigens in the context of MHC class I molecules, and IFNβ secretion needed for IFNAR1 signaling in CD8+ T cells. Thus, endothelial cells take a pivotal role in initiating adaptive immune responses that damage the BBB and lead to life-threatening brain edema.

On other hand, autonomous Type 1 IFN signaling is also involved in weakening intercellular junctions of endothelial cells. This effect was associated with disturbances in the Wnt/*ß*-catenin signaling pathway. It has been reported that in human brain endothelial cells, *ß*-catenin activation upon exposure to *Plasmodium falciparum*-IE was connected with angiotensin II signaling pathways (42). We found that Type 1 IFN signaling-dependent disturbances in the Wnt/*ß*-catenin system, were associated with endothelial junction dysfunction. Together these observations suggest a role for the Wnt/*ß*-catenin system in both ECM and human CM pathogenesis providing initial evidence of an autocrine/paracrine mechanism leading to dysfunction of the endothelial barrier acting in absence of systemic components. Such a mechanism would allow extravasation of intravascular fluid to the perivascular space, a critical step for vasogenic brain edema that is a frequent cause of death in subjects with cerebral malaria (41, 43).

It is well known that immunoproteasome induction acts in the processing of cytosolic antigens required for their presentation in the context of MHC I molecules (44). We found that immunoproteasome gene expression was induced in BECs exposed to *PbA-*IE. Such immunoproteasome activation likely contributes to the observed enhancement of antigen presentation efficiency in corroboration with previous findings that *Plasmodium* antigens are processed *via* a cytosolic pathway rather than the vacuolar pathway (15, 45). The *ß*-catenin cytoplasmic pool is tightly regulated *via* the ubiquitin-proteasomal pathway (19, 46) and we found that gene expression driven by Wnt/*ß*-catenin in BECs exposed to *PbA*-IE is partially recovered when the immunoproteasome is inhibited. Likewise, proteome analysis shows that the expression of several proteins related to cytoskeleton organization and intercellular junctions was altered by Type 1 IFN signaling. This suggests that immunoproteasome activation induced by Type 1 IFN signaling indirectly affects the stability of endothelial intercellular junctions which partially operate under the control of Wnt/*ß*-catenin signaling.

Moreover, we show that immunoproteasome induction in BECs was dependent on Type 1 IFN signaling but in contrast to established mechanisms did not require exogenous IFN-γ (47). This unexpected finding is only paralleled by observations that during hepatitis C virus infection immunoproteasome induction in the liver occurs in a Type I IFN-dependent manner (48). The precise mechanisms of immunoproteasome induction by Type 1 IFN are still unclear but further research may shed light on how Type 1 IFN operates in other non-immune cells to engage adaptive immune responses.

We found that IFNβ secretion in *PbA-IE* exposed BECs was preceded by and dependent on increased glucose consumption. Likewise, the blockade of glycolysis affected the downstream effects of Type 1 IFN signaling including, immunoproteasome induction, enhancement of antigen presentation, and down-regulation of Wnt signaling gene expression. It is widely accepted that in response to inflammation, cells shift glucose metabolism toward glycolysis (49, 50). Multiple pathway analysis uncovered an impact in glucose metabolism in *PbA-IE* exposed BECs. However, further studies using specific methodologies such as isotope tracer analysis of metabolic fluxes are required to establish the precise glucose metabolism shifts induced by *PbA-IE* exposure. Remarkably, the sharp increase in glucose uptake in BECs upon exposure to IE did not depend on host extra-endothelial inflammatory or metabolic stimuli. The IE components and interaction events that lead to increased glucose uptake in BECS remain to be identified. Our results also indicate that increased glucose uptake elicited by *Pb*A-IE infection is critical to the induction of IFNAR1 signaling in BECs that in turn precipitate pathogenesis mechanisms operating in the initiation of clinical ECM in the mouse (13). This suggest that metabolic adaptation in BECs is an ECM pathogenesis component, possibly not operating in non-cerebral models of malaria infection.

Overall, this work provides evidence that Type 1 IFN response in BECs exposed to IE is preceded by glucose metabolism alterations and is a driver of multiple effector mechanisms that likely underlie the main determinants of ECM fatality, namely brain immunopathology, BBB dysfunction and vasogenic edema.

## Data availability statement

The datasets presented in this study can be found in online repositories. The names of the repository/repositories and accession number(s) can be found below: PXD039805 (ProteomeXchange).

## Ethics statement

The animal study was reviewed and approved by Instituto Gulbenkian de Ciência Ethics Committee (Project number A05.2020).

## Author contributions

AS: Performed the experiments, analysed data, wrote the paper. AV: Performed proteome experiments and analysed the data. RZ: Analysed proteome data. CP-G: Conceived the hypothesis, analysed data, wrote the paper. All authors contributed to the article and approved the submitted version.
