# Corrigendum: Brain endothelial cells exposure to malaria parasites links type I interferon signalling to antigen presentation, immunoproteasome activation, endothelium disruption, and cellular metabolism

**DOI:** 10.3389/fimmu.2023.1331366

**Published:** 2023-11-28

**Authors:** Abdul Muktadir Shafi, Ákos Végvári, Shanshan Wu Howland, Roman A. Zubarev, Laurent Rénia, Carlos Penha-Gonçalves

**Affiliations:** ^1^ Disease Genetics, Instituto Gulbenkian de Ciência, Oeiras, Portugal; ^2^ Proteomics Biomedicum, Division of Physiological Chemistry I, Department of Medical Biochemistry and Biophysics, Karolinska Institutet, Stockholm, Sweden; ^3^ Division of Physiological Chemistry I, Department of Medical Biochemistry and Biophysics, Karolinska Institutet, Stockholm, Sweden; ^4^ Singapore Immunology Network, Agency for Science, Technology, and Research (A*STAR), Singapore, Singapore; ^5^ A*STAR Infectious Diseases Labs, Agency for Science, Technology, and Research (A*STAR), Singapore, Singapore; ^6^ Lee Kong Chian School of Medicine, Nanyang Technological University, Singapore, Singapore

**Keywords:** cerebral malaria (CM), type - 1 interferons, antigen presentation, endothelial cells, glucose metabolic alterations, proteome, metabolome, malaria

In the published article, there was an error in the author list, and authors Shanshan Wu Howland and Laurent Rénia were erroneously excluded. The corrected author list appears above.

In the published article, there was an error in the Funding statement as a funding source (SymbNET Genomics and Metabolomics in a Host-Microbe Symbiosis Network) was not included. The correct Funding statement appears below.

