# Supplementary material for: Brain endothelial cells exposure to malaria parasites links type I interferon signalling to antigen presentation, immunoproteasome activation, endothelium disruption, and cellular metabolism
Source: Front Immunol. 2023 Mar 13;14:1149107. doi: 10.3389/fimmu.2023.1149107 (PMC10042232; doi:10.3389/fimmu.2023.1149107)
Supplement: Supplementary file 1 [file DataSheet_1.pdf]

Brain Endothelial Cells Exposure to Malaria Parasites Links Type I Interferon Signalling to Antigen Presentation, Immunoproteasome Activation, Endothelium Disruption, and Cellular Metabolism

By

Abdul Muktadir Shafi, Ákos Végvári, Roman A Zubarev
and Carlos Penha-Gonçalves

SUPPLEMENTAL INFORMATION

SUPPLEMENTAL TABLES

Table S1: Differential protein expression in BECs from IFNAR1 KO and wild-type mice upon *in vitro* exposure to *PbA*-IE. Functional grouping of selected differentially expressed protein features.

Grouping	Protein	Feature ID	log2(FC)	-log10(p)	Obs
**Antigen presentation**					
	DC-STAMP domain-containing 2	A0A140LIJ0	-0.57	2.40	
	H-2 class I histocompatibility antigen, L-D alpha chain	P01897	-0.26	2.48	
	Legumain	O89017	0.69	3.01	
**Protein trafficking and folding**					
	Multiple coagulation factor deficiency protein 2 homolog	Q8K5B2	-0.55	2.93	
	Prefoldin subunit 3	Q3TIR6	-0.33	2.32	2 features
	Calumenin	Q6XLQ8	-0.33	2.61	2 features
	GTPase HRas	C0H5X4	0.26	2.33	
	Vacuolar protein sorting-associated protein 26A	A0A1W2P7Z9	0.37	2.08	
**Vesicle trafficking**					
	Protein S100-A10	P08207	-0.20	2.68	
	Y-box-binding protein 1	P62960	-0.19	2.70	
	Ras-related protein Rab-18	P35293	0.13	2.62	
	Protein transport protein Sec31A	Q3UPL0	0.19	2.87	
	Annexin A6	F8WIT2	0.21	2.52	2 features
	Protein transport protein Sec31A	S4R2A9	0.23	3.12	
	EH domain-containing protein 2	Q8BH64	0.32	2.38	
**Cytoskeleton/cell shape and motility**					
	WD repeat-containing protein 1	A0A0J9YU05	-0.52	3.48	
	Myosin regulatory light polypeptide 9	Q9CQ19	-0.50	2.35	
	Actin, gamma-enteric smooth muscle	A0A0U1RQ96	-0.45	2.74	
	Transgelin	A0A1L1STN8	-0.45	2.59	2 features
	Myristoylated alanine-rich C-kinase substrate	P26645	-0.40	2.79	
	Vimentin	P20152	-0.29	2.69	
	Myosin light polypeptide 6	Q60605	-0.27	2.72	4 features
	laminin subunit alpha-4	P97927	0.29	2.20	
	Laminin subunit beta-2	Q61292	0.45	2.33	
**Extracellular matrix/cell adhesion**					
	Zyxin	A0A0N4SVD2	-0.54	3.07	3 features
	Galectin-1	P16045	-0.35	2.86	
	PDZ and LIM domain protein 5	A0A0G2JEJ0	-0.30	3.00	
	Phosphoglucomutase-like protein 5	Q8BZF8	0.28	2.75	
	Ras-interacting protein 1	Q3U0S6	0.30	2.28	
	Afadin	A0A668KLE6	0.36	2.09	
	Heparan Sulfate Proteoglycan 2	E9PZ16	0.36	2.80	3 features
	Serine protease HTRA1	Q9R118	0.41	2.31	
	Thrombospondin-1	P35441	0.45	3.13	
**Metabolism**					
	ATP synthase, H+-transporting, F1 complex		-0.30	2.01	7 features
	Adenine phosphoribosyltransferase		-0.40	2.08	
	Adenosylhomocysteinase		0.29	3.34	

FC represents KO/wild-type relative abundance. Shown are protein features with P value <0.01 (-log10(p)<2) and log2(FC) > 0.18, red; log2(FC) < - 0.18, blue. For proteins with more than one significant feature, the feature with largest FC effect is shown.

Proteins identified from respective feature ID in the SwissProt database. FC represents KO/wild-type rate. Shown are protein features with P value <0.01 (-log10(p)<2) and log2(FC) > 0.18 or < - 0.18. Proteins with higher expression in wild-type (FC <0) in blue and with lower expression (FC>0) in red. For proteins with more than one differentially expressed feature, statistics refer to feature with largest FC effect is shown.

SUPPLEMENTAL TABLES

Table S2: Change in metabolites content in BECs exposed to *PbA*-IE detected in multiple targeting pathway analysis.

Metabolite	p value	FC (exposed /unesposed)
GLUTARATE	7.48E-05	2.79
PROPIOYLCARNITINE	3.76E-03	2.28
DEOXYRIBOSE	4.38E-03	1.84
OXOPROLINE	2.47E-02	1.66
ISOLEUCINE	3.56E-02	1.58
4-ACETAMIDOBUTANOATE	9.09E-03	1.53
PANTOTHENATE	3.55E-02	1.44
CREATINE	1.96E-02	1.39
LACTATE	4.53E-02	1.37
ALANINE	8.95E-03	1.36
N,N,N-TRIMETHYLLYSINE	2.94E-02	1.32
HEXANOYLCARNITINE	2.64E-02	1.31
CIS-ACONITATE	3.95E-02	1.31
DIMETHYLARGININE (SDMA/ADMA)	3.96E-02	1.29
BUTYRYLCARNITINE	2.25E-02	1.28
BETAINE	3.97E-02	1.19
ASPARTATE	2.43E-02	1.13
THREONINE	3.06E-02	0.81
INOSINE MONOPHOSPHATE	2.12E-02	0.69

P values of t-tests ($P<0.05$) and fold change (FC) comparing groups of three samples of exposed versus unexposed cells.

SUPPLEMENTAL FIGURE LEGENDS

Figure S1. Claudin 5 gene expression in BECs is down-regulated upon exposure to *Pb*A-IE.

Gene expression of *Cldn5* quantified in wild-type **(A)** and IFNAR1 KO BECs **(B)** at indicated time-points BECs 24 after exposure to *Pb*A-IE in the presence or absence of immunoproteasome inhibitor ONX-0914 (300 nM)(ONX)**(C)**. Gene expression of *Cldn5* quantified in brains of uninfected (n=6) and infected (n=9) mice **(D)**. Results of relative quantification gene expression are represented as fold change ($2^{\Delta\Delta CT}$) using unexposed BECs or uninfected mice as controls. Statistics: Significant results of pairwise comparisons in ANOVA tests are shown (*; p<0.05, **; p<0.01, ***; p <0.001).

Figure S2. Effects of Glycolysis inhibition on antigen presentation of loaded peptides and expression of Wnt/*ß*-catenin target genes.

Effects of inhibition of glycolysis by incubation of BEC cultures with 2-DG (10 mM) measured 24 h after exposure or not to *Pb*A-IE. **(A)** Antigen presentation of cognate peptide (SQLLNAKYL) (10 µM) loaded *in vitro* is not impaired by incubation with 2-DG. Results of pairwise comparisons in ANOVA tests (*; p<0.05, ***; p <0.001). **(B)** Gene expression of *ß*-catenin target genes *Edn1*, *Axin2*, *Nkdd1*, and *Apcdd1* as well as *cldn5* was quantified in wild-type BECs 24 h after exposure to *Pb*A-IE in the presence (red bars) or absence (black bars) of 2-DG or in presence of 2-DG (blue bars) alone. Relative quantification of gene expression is represented as fold change ($2^{\Delta\Delta CT}$) using unexposed BECs as controls (dashed line). Statistics: Results of pairwise group comparisons for 2-DG effect in 2-way ANOVA tests (*; p<0.05, ****; p <0.0001).

Figure S3. Glycolysis blockade: BECs glucose consumption *in vitro* and parasitemia *in vivo*.

(A) Measurement of glucose in supernatants of BEC cultures exposed or not to *Pb*A-IE for 24 h in the presence or absence of 2-DG (10 mM). After 24-h exposure medium containing *Pb*A-IE was drained and cultures were replenished with fresh medium. Glucose measurements were performed 24 h after adding fresh medium. **(B)** Daily parasitemia measurements in individual mice infected with *Pb*A and treated (n=10) or not (n=9) with 2-DG injection (800 mg/kg) at day 4.

FIGURE S1

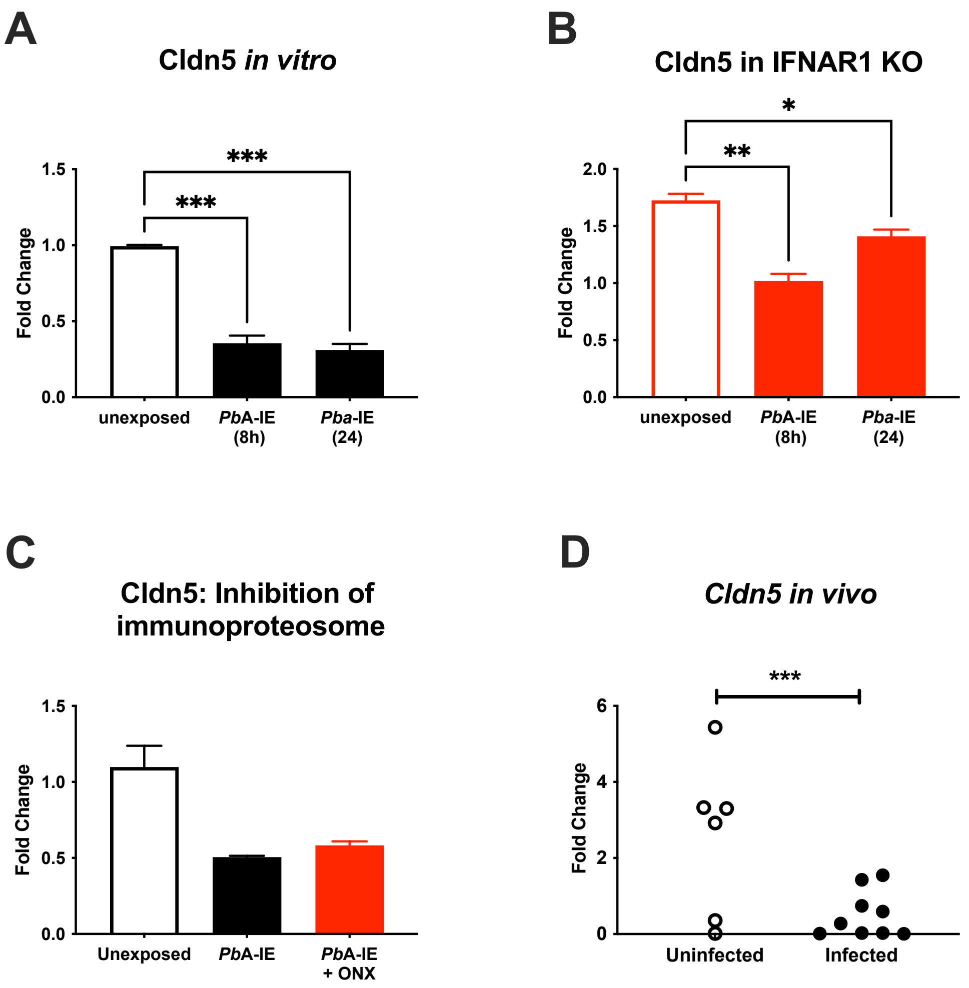
A
Cldn5 *in vitro*
***
***
1.5
1.0
0.5
0.0
Fold Change
unexposed
*Pb*A-IE (8h)
Pba-IE (24)
B
Cldn5 in IFNAR1 KO
*
**
2.0
1.5
1.0
0.5
0.0
Fold Change
unexposed
*Pb*A-IE (8h)
Pba-IE (24)
C
Cldn5: Inhibition of immunoproteosome
1.5
1.0
0.5
0.0
Fold Change
Unexposed
*Pb*A-IE
*Pb*A-IE + ONX
D
Cldn5 in vivo
***
6
4
2
0
Fold Change
Uninfected
Infected

FIGURE S2

A

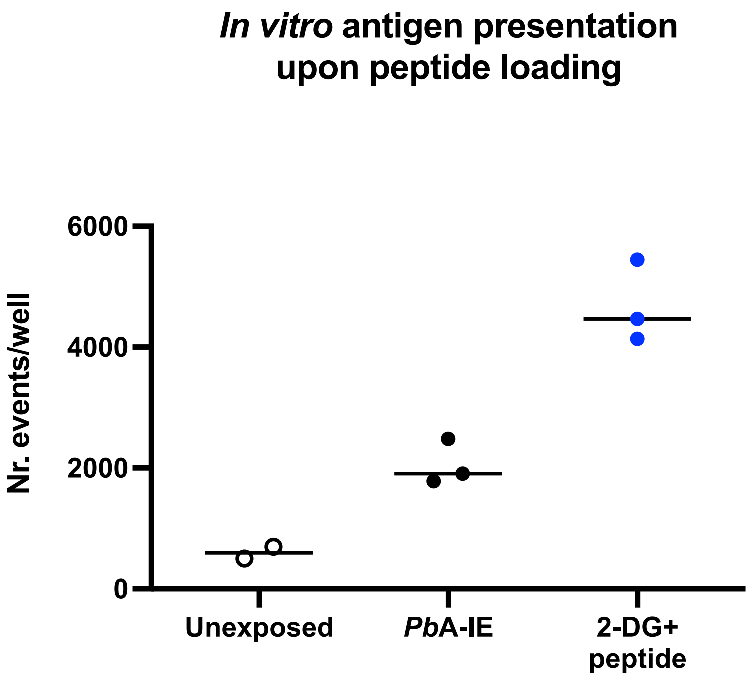
In vitro antigen presentation
upon peptide loading
Nr. events/well
6000
4000
2000
0
Unexposed
*Pb*A-IE
2-DG+
peptide

B

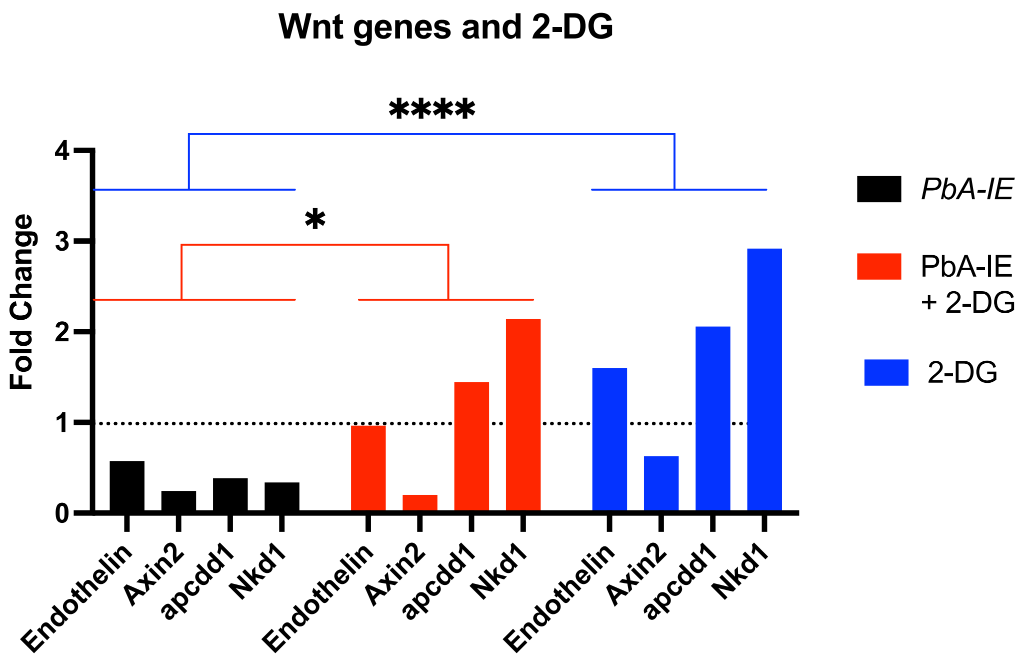
Wnt genes and 2-DG
****
*
Fold Change
4
3
2
1
0
Endothelin
Axin2
apcdd1
Nkd1
Endothelin
Axin2
apcdd1
Nkd1
Endothelin
Axin2
apcdd1
Nkd1
PbA-IE
PbA-IE
+ 2-DG
2-DG

FIGURE S3

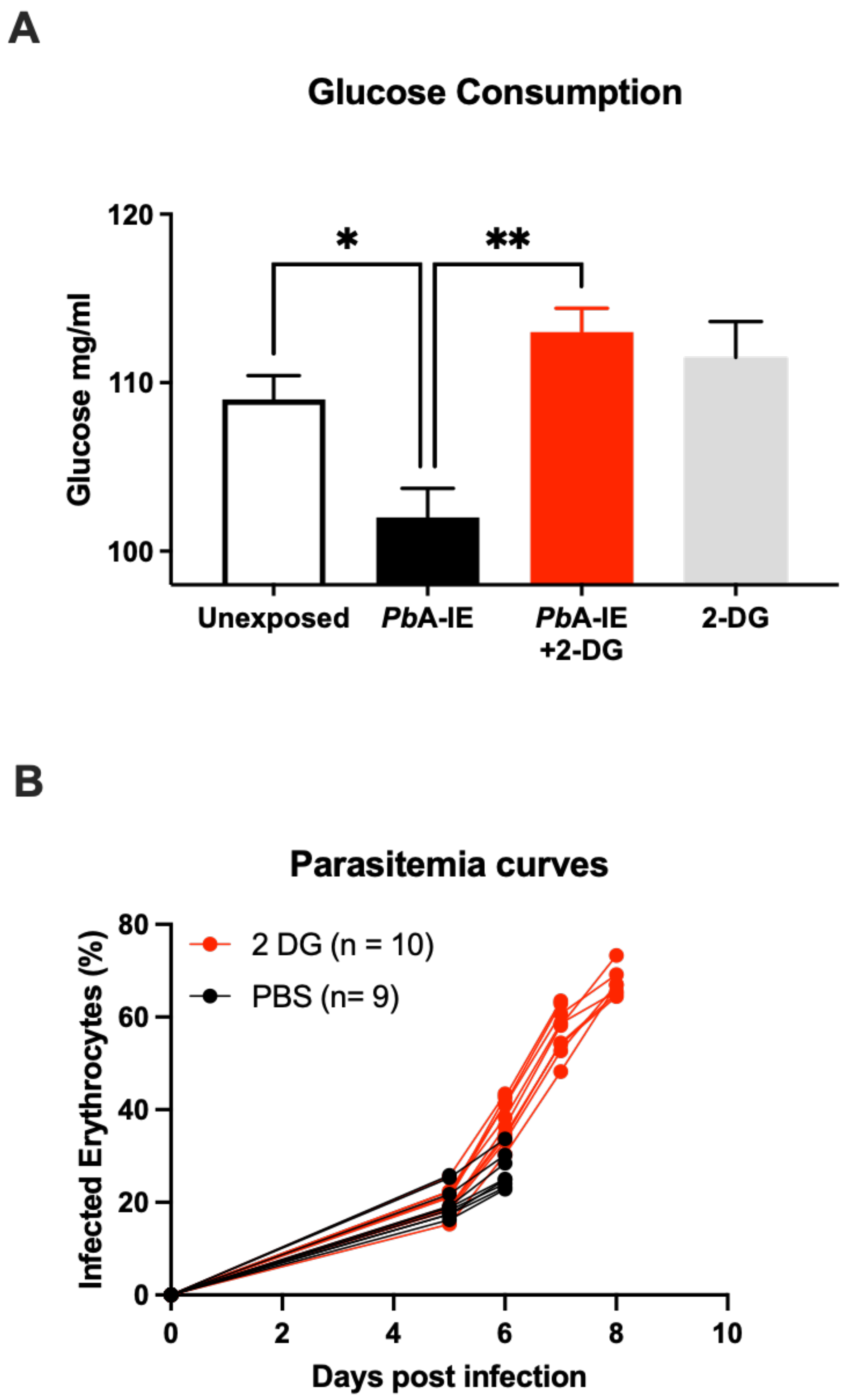
A
Glucose Consumption
Glucose mg/ml
120
110
100
*
**
Unexposed
PbA-IE
PbA-IE
+2-DG
2-DG
B
Parasitemia curves
Infected Erythrocytes (%)
80
60
40
20
0
2 DG (n = 10)
PBS (n= 9)
0
2
4
6
8
10
Days post infection
